# Vascular Organoids Derived from Capillary malformation-induced Pluripotent Stem Cells Exhibit Disease-Relevant Phenotypes

**DOI:** 10.1007/s12015-025-10984-8

**Published:** 2025-09-25

**Authors:** Vi Nguyen, Anna Harper, Mackenzie Azuero, Isabella Castellanos, Siwuxie He, Marcelo L. Hochman, Camilla F. Wenceslau, Dong-bao Chen, Anil G. Jegga, Yunguan Wang, Daping Fan, J. Stuart Nelson, Wenbin Tan

**Affiliations:** 1 Department of Cell Biology and Anatomy, School of Medicine, University of South Carolina, Columbia, SC 29209 USA; 2 The Facial Surgery Center and the Hemangioma & Malformation Treatment Center, Charleston, SC 29425 USA; 3 Department of Otolaryngology - Head and Neck Surgery, Medical University of South Carolina, Charleston, SC 29425 USA; 4 Department of Biomedical Engineering, College of Engineering and Computing, University of South Carolina, Columbia, SC 29208 USA; 5 Department of Obstetrics and Gynecology, University of California, Irvine, CA 92617 USA; 6 Department of Pediatrics, University of Cincinnati College of Medicine, Cincinnati, OH 45229 USA; 7 Division of Biomedical Informatics, Cincinnati Children Hospital Medical Center, Cincinnati, OH 45229 USA; 8 Departments of Surgery and Biomedical Engineering, Beckman Laser Institute and Medical Clinic, University of California, Irvine, CA 92617 USA; 9 Division of Pediatric Gastroenterology, Hepatology and Nutrition, Cincinnati Children Hospital Medical Center, Cincinnati, OH 45229 USA

**Keywords:** Capillary Malformation, Human Induced Pluripotent Stem Cells, Vascular Organoid, Endothelial Cells, Smooth Muscle Cells

## Abstract

Capillary malformation (CM) is a congenital vascular anomaly that affects the skin, mucosa, eye, and brain. A major obstacle to mechanistic and drug screening studies for CM has been the lack of preclinical models. In this study, we established vascular organoids (VOs) generated through the self-assembly of vascular lineages of endothelial cells and smooth muscle cells differentiated from CM-induced pluripotent stem cells (iPSC). Within these VOs induced endothelial cells and smooth muscle cells organized into juxtapositions to form vascular branches. CM patient iPSC-derived VOs showed a higher density of endothelial and smooth muscle cell populations and greater vascular branch lengths as compared with VOs derived from iPSCs generated from healthy skin biopsies. Overall, this study represents the first disease-relevant VO model of CM, providing a valuable platform for future mechanistic studies and drug screening.

**Supplementary Information:**

The online version contains supplementary material available at 10.1007/s12015-025-10984-8.

## Introduction

Congenital vascular malformations are developmental vasculature defects, occurring in a 1.5% of general population [1]. The ISSVA categorizes several subtypes of congenital capillary malformations (CMs), including cutaneous and/or mucosal CM (a.k.a., port-wine birthmarks, PWB), leptomeningeal CM with brain involvement (Sturge-Weber syndrome, SWS), diffuse CM with overgrowth, reticulate CM, and some subtypes of telangiectasia [2]. Cutaneous CM is the most common type of vascular malformations with a prevalence of 0.3%–0.5% of live births [3]. In particular, around 25% of CM infants with forehead lesions have an increased risk of SWS that presents ipsilateral leptomeningeal angiomatosis [4]. Cutaneous CM appears as flat red macules in childhood, tend to progressively darken to purple, and often become raised as vascular nodules which are susceptible to spontaneous bleeding or hemorrhage [3]. CM lesions typically exhibit various vascular phenotypic remodeling, including differentiation impairments, proliferation of endothelial cells (ECs) and smooth muscle cells (SMCs), replication of vascular basement membranes, disruption of vascular barriers, and progressive dilatation of vasculature [5]. However, the underlying mechanisms are incompletely understood.

Currently, the absence of preclinical cell and animal models remains a critical barrier hampering the development of mechanistic and drug screening studies for CM. We recently generated induced pluripotent stem cells (iPSCs) from CM biopsies and differentiated their induced ECs (iECs) to overcome this obstacle [6]. The CM iECs have been shown to preserve many lesion-like phenotypes, including forming enlarged vasculature in vitro and in vivo [6]. In the current study, we here for the first time reported a generation of vascular organoids (VOs) through self-assembly of multiple vascular lineages derived from CM iPSCs.

##  Materials and Methods

### IPSC Culture

The generation and characterization of iPSC lines from normal and CM skin biopsies were reported previously [6, 7]. Briefly, surgically excised nodular CM lesions from two patients (#3921 and #4221) and one age- and sex-matched deidentified surgically discarded normal skin tissue (#52521) were collected, followed by the outgrowth and reprogramming of human dermal fibroblasts to generate CM and normal iPSCs. Both patients were Caucasians, male, 40–50 years old, had hypertrophic and nodular lesions, and received multiple rounds of pulsed dye laser treatments prior to surgical procedures. One patient had large lesions on his back, chest, arm and hand. The other patient had lesions on face [6, 7]. For monolayer culture, the iPSCs were maintained and propagated under feeder-free conditions using Essential 8 Medium (ThermoFisher, Waltham, MA) or mTeSR (StemCell Technologies, Vancouver, BC, Canada) on a Geltrex-coated plate. When iPSC confluence reached to 80%, cell dissociation was performed using enzyme-free Gentle Cell Dissociation Reagent (StemCell Technologies, Vancouver, BC, Canada). For a monolayer culture, the cells were then passed in a ratio of 1:6 with addition of Rock inhibitor Y27632 (10 µM) during the first 24 h. The refresh Essential 8 or mTeSR medium will be replaced every other day.

### VO Induction

Monolayer normal and CM iPSCs were dissociated and seeded into a 96 U-shape low-binding bottom plate with a density of 6000 cells in 100 µl of StemScale medium (ThermoFisher, Waltham, MA) using a higher concentration of Y27632 (20µM) for spheroids culture that have multiple cell layer assembled. The next day, 100 µl of StemScale medium was added to each well. At this stage, multiple small spheroids at the bottom of the well could be observed. On day 3, the medium was replaced with 100 µl of mesodermal induction medium (DMEM/F12 with 100ng/mL Activin A, 1X B27 without insulin, 1X N2 supplement, 1X Glutamax, 50µM β-mercaptoethanol, and 0.1X geltrex). On day 4, the medium was replaced with 100 µl medium of DMEM/F12, 30ng/mL BMP4, 10ng/mL bFGF, 50ng/mL VEGF, 1X N2 supplement, 1X Glutamax, 50µM β-mercaptoethanol, 1µM CHIR, and 0.1X Geltrex. On day 6, the medium was replaced with 100 µl DMEM/F12 containing 30ng/mL BMP4, 10ng/mL bFGF, 100ng/mL VEGF, 1X B27 without insulin, 1X N2 supplement, 1X Glutamax, and 50µM β-mercaptoethanol. On day 7, the medium was changed to 100 µl EC 1 st induction medium (StemPro complete medium containing 1X Glutamax, 200ng/mL VEGF, 200µM 8-Br-cAMP, 2µM Forskolin, and 10µM SB431542). The VO was embedded on day 9. The embedding material included Matrigel for organoid, 2% Collagen I, 100ng/mL b-FGF, 100ng/mL of VEGF, 2 µg/mL of Fibronectin, and 2 µg/mL of Heparin. Embedded VO was put in 700 µl EC 2nd induction medium (StemPro complete medium containing 20% FBS, 200ng/mL VEGF, 200µM 8-Br-cAMP, and 30ng/mL of b-FGF) in 24-well ultralow binding well plate. The medium was replaced half with the EC 2nd induction medium every other day until day 21.

### Whole Mount IF Staining

The VOs were collected and fixed in 2% paraformaldehyde overnight. The VOs were permeabilized using 0.1 M Glycine in phosphate-buffered saline with 0.2% Triton-X (PBST) overnight and blocked with 5% BSA in PBST for 2 h. The VOs were further blocked with 10% Goat Serum in PBST 4 h. The primary antibodies were diluted in 1% BSA in PBST and incubated with VOs at 4 °C for 48 h. The resources of primary antibodies were as follows: CD144 (VE-cadherin, CDH5) (monoclonal Antibody 16B1, Alexa Fluor™ 488, eBioscience™, Cat# 53-1449-42, 1:100 dilution) to label iECs, CoraLite^®^ Plus 647-conjugated SMA specific Recombinant antibody (Proteintech, Cat# CL647-80008, 1:100 dilution) to identify induced SMCs (iSMCs), and UEA I (DyLight^®^ 594-conjugated, Vector Laboratories, Cat#DL-1067-1, 1:100 dilution) to recognize human iECs. The VOs were washed with 1% BSA in PBST 2 times, 30 min each, followed by washing with PBST 2 times, 30 min each. The VOs were then stained with DAPI solution (1:1000) for 2 h and washed with PBS for 30 min, 3 times.

### Light Sheet Fluorescent Microscopy Imaging

VOs were embedded in 1.5% ultra-pure agarose inside 1.9 mm diameter glass capillaries and incubated overnight at room temperature in X-CLARITY mounting solution prior to imaging. Then the organoids were suspended in X-CLARITY clearing solution with a refractive index (RI) of 1.44 within the Zeiss Lightsheet 7 imaging chamber. Imaging was performed using 405, 488, 561, and 638 nm lasers with dual-side structured illumination and pivot scanning to enhance optical sectioning and reduce shadowing artifacts. An EC Plan-NEOFLUAR 5x/0.16 focusable objective and 5x/0.1 focusable illumination objectives were used to capture high-resolution volumetric data. Z-stack acquisition covered a thickness of approximately 1.7 mm, ensuring comprehensive imaging of the organoids. The acquired z-stacks were then processed and combined into a 3D reconstruction using Zeiss ZEN analysis software.

For quantitative analysis, 4–8 cross sections in different Z-positions per VO (*n* = 3 VO per group) were used for cell density (per mm^2^) counting and vascular branch length measurement. The binary images of each cross-section of VOs were used. The skeleton plugin in Image J was used to measure the branch length for each assigned skeleton structure.

## Statistical Analysis

Paired samples t-test or Mann-Whitney U test was performed to evaluate the statistical differences between CM and normal control data sets for cell density or vascular branch length, respectively. Data presented as “mean ± S.D.” or median (IQR), and *p* < 0.05 were considered significant.

## Results

**Fig. 1:**
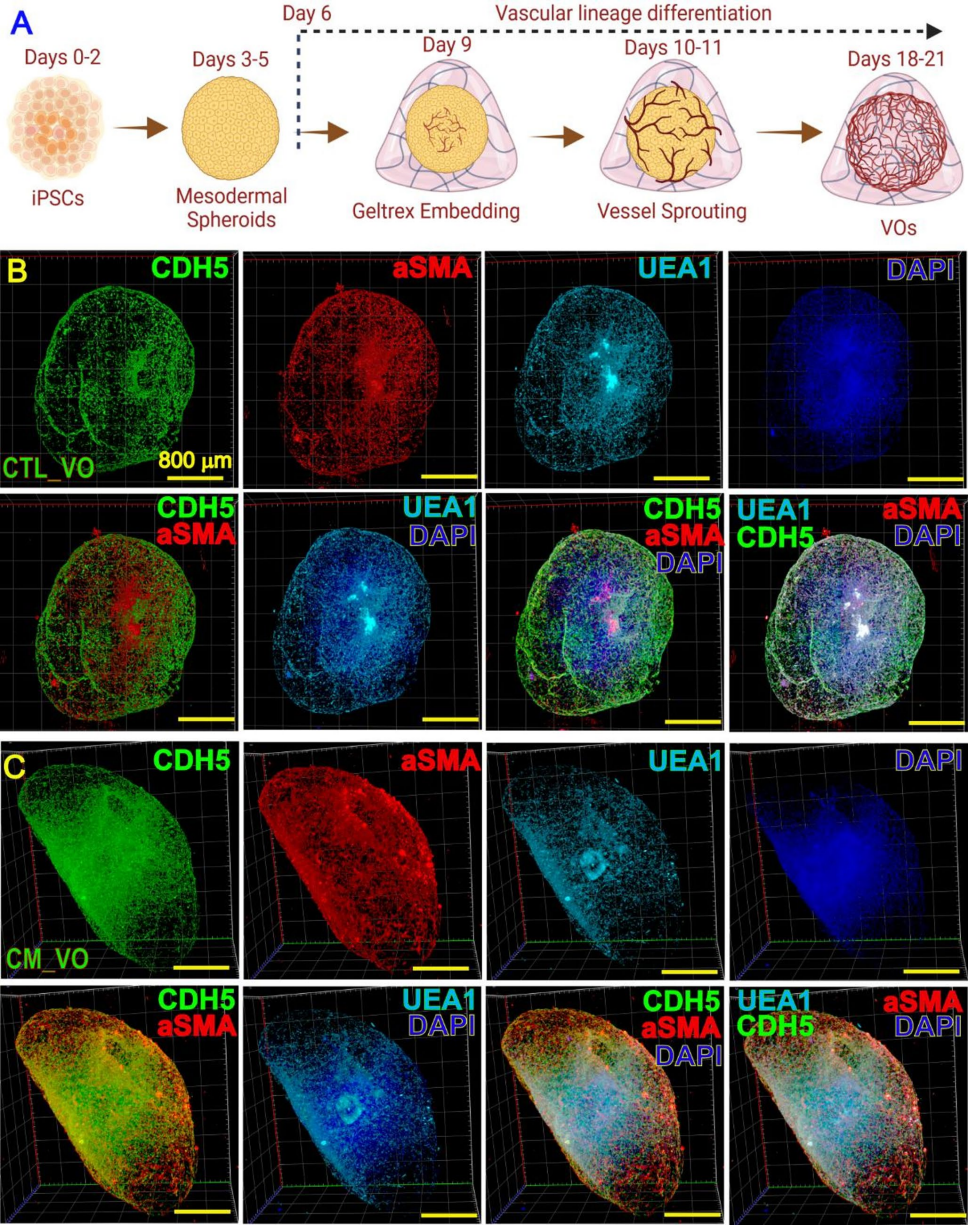
VOs induction from normal and CM iPSCs. A: Schematic of VO differentiation protocol. B and C: Whole mount IF staining was performed to show 3D structures of control (**B**) and CM VO (**C**) using an anti-CDH5 (a.k.a. CD144) (green), anti-UEA1 (light blue), and anti-aSMA (red) antibody. Images were acquired using Zeiss light sheet fluorescence microscopy and presented in maximum intensity projection mode (Zeiss Zen software). DAPI staining was used to reveal nuclei (dark blue). Overlay images of CDH5/aSMA, UEA1/DAPI, UEA1/CDH5/DAPI, and UEA1/CDH5/aSMA/DAPI were shown in each panel

For VO induction, normal and CM iPSCs were first cultured to form spheroids, followed by induction into mesodermal cell spheroids and embedded into Geltrex for vascular lineage induction with VEGF, FGF, 8-Br-cAMP, and Forskolin (Fig. 1A). The vascular sprouting could be observed within 24–48 h post spheroid embedding. The vascular network formation and maturation within VOs were usually completed in ~ 3 weeks upon induction. The VOs were stained using anti-CDH5, anti-UEA1, and anti-αSMA antibodies and overall VO architectures were imaged using light sheet fluorescence microscopy (Figs. 1B and C).

**Fig. 2:**
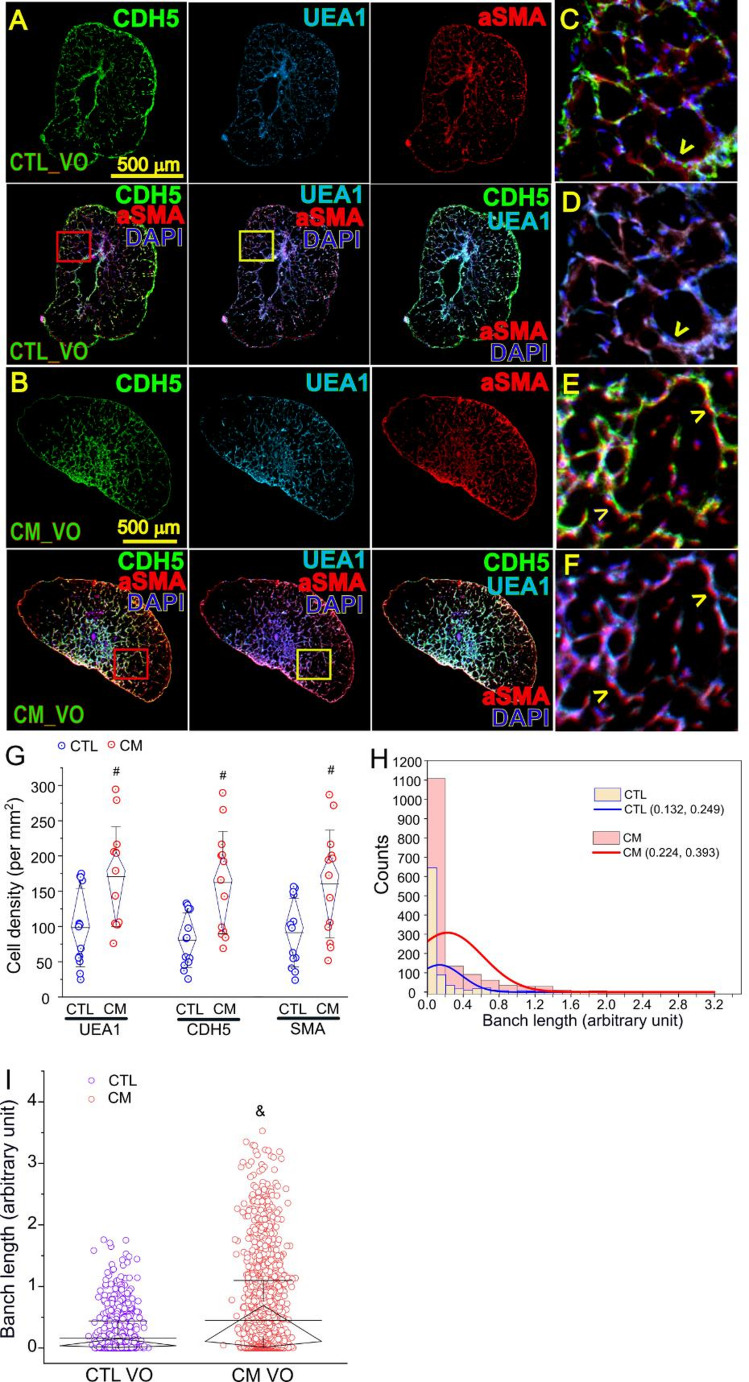
Structural characterizations of CM VOs. A and B: Cross sections of control (**A**) or CM (**B**) VO with IF staining of CDH5 (green), UEA1 (light blue), aSMA (red), or overlay of CDH5/aSMA/DAPI, UEA1/aSMA/DAPI, and CDH5/UEA1/aSMA/DAPI. C-F: higher magnifications of red (**C**, **E**) or yellow (**D**, **F**) boxed areas from (**A**) and (**B**), respectively, showing juxtapositions of iECs and iSMCs (yellow arrowheads) in vascular branches. G: UEA1, CDH5 or aSMA positive cell density (per mm^2^) in control and CM VOs. H: Distribution of vascular branch length in arbitrary unit. I: Arbitrary vascular branch length in control and CM VOs. #, *p* < 0.005 as compared to the control group, paired *t*-test; &, *p* = 1.56 × 10^−33^ as compared to the control group, Mann-Whitney U test. *N* = 3 VOs per group and 4–8 cross sections for (**G**) and (**I**) per VO

Both iECs and iSMCs were observed in VOs (Figs. 2A and B). Many iECs (CDH5^+^ or UEA1^+^) and iSMCs (aSMA^+^) were organized into juxtapositions to form vascular networks (Figs. 2C-F). The cell densities of iEC and iSMC (per mm^2^) in CM VOs were significantly higher as compared to normal VOs [UEA1 iECs: 92.4 ± 53.6 (CTL) versus 172.9 ± 73.6 (CM); CDH5 iECs: 76.8 ± 37.8 (CTL) versus 163.8 ± 75.5 (CM); aSMA: 86.4 ± 47.6 (CTL) versus 161.8 ± 72.6 (CM); *p* < 0.005; Fig. 2G]. The vascular branch lengths in CM VOs were greater as compared to normal VOs [median (IQR) in arbitrary unit: 0.036 (0.008, 0.153) (CTL) versus 0.106 (0.014, 0.693) (CM); Figs. 2H and I]. The consistency of vascular phenotypes across multiple VOs was shown in cross-sections in Z position-series from CTL and CM VOs (Supplementary Fig. 1).

We next examined whether our established CM iPSC lines carry known CM-related somatic mutations such as guanine nucleotide-binding protein alpha subunit q (GNAQ), phosphatidylinositol 3-kinase catalytic subunit alpha (PI3KCA), and mitogen-activated protein kinase kinase kinase 3 (MAP3K3) [8–12]. We performed bulk RNAs-seqs on these iPSCs and iECs. We find that these cells do not harbor known mutations including GNAQ (R183Q), GNAQ (Q209L), GNAQ (Q209R), PI3KCA (E542K), PI3KCA (E545K), PI3KCA (E547K), PI3KCA (E1037G), PI3KCA (H1047R), MAP3K3 (L114I), and MAP3K3 (I441M) (Table 1).

**Table 1:** The targeting SNV frequency among iPSC lines by bulk RNA-seq

		GNAQ			MAP3K3		PIK3CA				
		548G > A (R183Q); chr9:77,797,577	626 A > T (Q209L); chr9:77,794,572	626 A > G (Q209R); chr9:77,794,572	658 C > A (L114I); chr17:63,652,636	1323 C > G (I441M); chr17:63691212	1624G > A (E542K); chr3:179,218,294	1633G > A (E545K); chr3:179,218,303	1639G > A (E547K); chr3:179,218,309	3110 A > G (E1037G); chr3:179,234,268	3140 A > G (H1047R); chr3:179,234,300
IPSC line	Disease	SNV/total counts	SNV/total counts	SNV/total counts	SNV/total counts	SNV/total counts	SNV/total counts	SNV/total counts	SNV/total counts	SNV/total counts	SNV/total counts
3921_9	CM	0/136	0/168	0/168	1/98	0/109	0/63	0/71	0/72	0/64	0/65
3921_16	CM	0/139	0/161	0/161	1/55	0/47	0/64	0/69	0/68	0/60	0/68
3921_1	CM	0/95	0/125	0/125	0/27	0/43	0/63	0/66	0/65	0/64	0/63
4221_3	CM	0/145	0/186	0/186	0/42	0/64	0/47	0/47	0/48	0/44	0/39
4221_6	CM	0/156	0/208	0/208	0/61	0/91	0/76	n.a.	n.a.	0/54	0/164
52521_8	Normal	0/150	0/174	0/174	0/48	0/64	0/49	n.a.	n.a.	0/62	0/68
52521_4	Normal	0/50	0/70	0/70	0/127	0/38	0/37	n.a.	n.a.	0/89	0/11
52521_9	Normal	0/152	0/195	0/194	0/90	0/126	0/112	n.a.	n.a.	0/32	0/30

## Discussion

Sporadic somatic mutations of GNAQ (R183Q) and PI3KCA have been linked to the vascular phenotypes of CM [8, 9]. For example, GNAQ (R183Q) has allelic frequencies ranging from 1 to 12% (about 4.7% on average) in skin lesions [8]. GNAQ (R183Q) is present in ECs or blood vessels [10, 13], connective tissues, hair follicles, and glands in CM lesions, suggesting that pluripotent cells with GNAQ (R183Q) give rise to multiple lineages in cutaneous CM [10]. EPCs with endogenous wild type GNAQ and forced-expression of GNAQ (R183Q) can cause active phospholipase C β3 signaling, increase angiopoietin-2, and produce enlarged capillary-like vessels [12]. In a mouse model, the GNAQ p.R183Q mutation was introduced by crossing with either mosaic global E2a- Cre mice or ubiquitous global β-actin-Cre mice [14]. The results showed that mice with the global β-actin-Cre experienced complete embryonic lethality, whereas those with the mutated mosaic global E2a-Cre exhibited partial embryonic lethality [14]. Examination of the embryos at E13.5 to 14.5 revealed dilated vascular channels and edema, suggesting the presence of vascular defects [14]. In another example, mice with the GNAQ p.Q209L mutation under the CMV-Cre promoter displayed earlier lethality at E8.5 [15]. These findings demonstrate that homogenous inheritance of the GNAQ mutation leads to lethality rather than survival of CM ECs [14, 15]. However, CM patient-derived induced pluripotent stem cells (iPSCs) we have generated do not harbor the GNAQ (R183Q) mutations but inherit many clinically relevant vascular phenotypes in vitro and in vivo [6, 7]. One explanation is that CM iPSCs preserve critical epigentic signatures from origninal lesions for pathological recapitulation. It is unknown which types of lesional epigenetic patterns could have been carried over by the CM iPSCs and their derived iECs and VOs and what their roles are in vascular phenotype development. This will be our future study. In addition, evidence has shown the increased numbers of macrophages in the perivascular sites of CM blood vessels in brain, eye, and skin lesions [16, 17], suggesting that inflammatory components are important contributors to the pathological progression of CM. Proinflammatory signaling such as tumor necrosis factor alpha/ nuclear factor kappa Bhas been recapitulated by CM iPSCs [6], potentially acting as GNAQ (R183Q)-independent pathogenic factor.

There are several advantages of this VO model as compared with available models used for CM studies. First it represents many patient-specific phenotypes, thus serving as a clinically relevant model. Second, it is constituted with multiple vascular lineages in 3D architecture in a dish, providing an unprecedent in vitro model of the disease. Third, the data is generated from lesion-derived cells and structures in this model, thus it is translational. Fourth, it can be further integrated with specific disease-relevant genetic variants for mechanistic and drug screening studies. The limitations of this VO model and current study are as follows. First, other vascular related lineages or biological components are absent in the current model such as immune cells and axonal innervations. Second, there are potential biological defects of induced VOs such as containing iECs or iSMCs under different differential stages. Third, it can recapitulate only partial but not full phenotypes from lesions. Fourth, physiological functions such as flow and contractility in this model are yet to be established. Fifth, functional evaluations of this VO model, including endothelial barrier integrity and inflammatory responsiveness to external stimuli such as TNFα and hypoxia, have not been performed. This will be tested in our future studies.

In conclusion, we have generated CM iPSC-derived VO models that recapitulate many CM-like vascular phenotypes including a high density of vascular branches and an increased SMC population. These data demonstrate that the CM VOs are new and valuable disease-relevant models for mechanistic and drug screening studies.

## Electronic supplementary material

Below is the link to the electronic supplementary material.

Supplementary Material 1 (DOCX 1.91 MB)

## Data Availability

The RNA-seq data are deposited into NIH SRA (Sequence Read Archive) (BioProject ID: PRJNA997591) and the NCBI GEO (Gene Expression Omnibus) (Accession number: GSE240770). Data is available to the public.

## Abbreviations

bFGF: Basic Fibroblast Growth Factor
BMP4: Bone Morphogenetic Protein 4
CDH5: Cadherin 5, VE-cadherin or CD144
CM: Capillary Malformation
EC: Endothelial Cell
GNAQ: Guanine Nucleotide-binding Protein Alpha Subunit Q
iEC: induced Endothelial Cell
iPSC: Induced Pluripotent Stem Cell
IQR: Interquartile Range
ISSVA: The International Society for the Study of Vascular Anomalies
iSMC: induced Smooth Muscle Cells
MAP3K3: Mitogen-activated Protein Kinase Kinase Kinase 3
PBST: Phosphate-buffered Saline With Triton-X
PI3KCA: Phosphatidylinositol 3-kinase Catalytic Subunit Alpha
PWB: Port-wine Birthmarks
SMC: Smooth Muscle Cells
SWS: Sturge-Weber Syndrome
UEA I: Ulex Europaeus Agglutinin I
VEGF: Vascular Endothelial Growth Factor
VO: Vascular Organoid

## References

[1] Eifert, S., Villavicencio, J. L., Kao, T. C., Taute, B. M., & Rich, N. M. (2000). Prevalence of deep venous anomalies in congenital vascular malformations of venous predominance. *Journal of Vascular Surgery*, *31*(3), 462–471. 10709058

[2] ISSVA. (2018). *ISSVA classification of vascular anomalies*. International Society for the Study of Vascular Anomalies issva.org/classification.

[3] Lever, W. F., & Schaumburg-Lever, G. (1990). *Histopathology of the skin* (7th ed.). J.B. Lippincott Co.

[4] Waelchli, R., Aylett, S. E., Robinson, K., Chong, W. K., Martinez, A. E., & Kinsler, V. A. (2014). New vascular classification of port-wine stains: Improving prediction of Sturge-Weber risk. *British Journal of Dermatology*, *171*(4), 861–867. 24976116 10.1111/bjd.13203 PMC4284033

[5] Tan, W., Zakka, L. R., Gao, L., Wang, J., Zhou, F., Selig, M. K., et al. (2016). Pathological alterations involve the entire skin physiological milieu in infantile and early childhood Port wine stain. *British Journal of Dermatology*, *177*(1), 293–296. 10.1111/bjd.15068 PMC5793867 27639180

[6] Nguyen, V., Gao, C., Hochman, M. L., Kravitz, J., Chen, E. H., & Friedman, H. I. (2023). Endothelial cells differentiated from patient dermal fibroblast-derived induced pluripotent stem cells resemble vascular malformations of Port wine birthmark. *British Journal of Dermatology*. 10.1093/bjd/ljad330 37672656 10.1093/bjd/ljad330 PMC10653332

[7] Nguyen, V., Gao, C., Hochman, M. L., Kravitz, J., Chen, E. H., & Friedman, H. I. (2023). Supporting materials: Endothelial cells differentiated from patient dermal fibroblast-derived induced pluripotent stem cells resemble vascular malformations of Port Wine Birthmark. *bioRxiv*10.1093/bjd/ljad330 PMC10653332 37672656

[8] Shirley, M. D., Tang, H., Gallione, C. J., Baugher, J. D., Frelin, L. P., Cohen, B., et al. (2013). Sturge-weber syndrome and port-wine stains caused by somatic mutation in GNAQ. *New England Journal of Medicine,**368*(21), 1971–1979. 23656586 10.1056/NEJMoa1213507 PMC3749068

[9] Lian, C. G., Sholl, L. M., Zakka, L. R., O, T. M., Liu, C., Xu, S., et al. (2014). Novel genetic mutations in a sporadic port-wine stain. *JAMA Dermatology,**150*(12), 1336–1340. 25188413 10.1001/jamadermatol.2014.1244

[10] Tan, W., Nadora, D. M., Gao, L., Wang, G., Mihm, M. C. Jr., & Nelson, J. S. (2016). The somatic GNAQ mutation (R183Q) is primarily located in Port wine stain blood vessels. *Journal of the American Academy of Dermatology*, *74*(2), 380–383. 26775782 10.1016/j.jaad.2015.09.063 PMC5110233

[11] Zhang, B., He, R., Xu, Z., Sun, Y., Wei, L., Li, L., et al. (2023). Somatic mutation spectrum of a Chinese cohort of pediatrics with vascular malformations. *Orphanet Journal of Rare Diseases*, *18*(1), 261. 37658401 10.1186/s13023-023-02860-w PMC10474751

[12] Huang, L., Bichsel, C., Norris, A. L., Thorpe, J., Pevsner, J., Alexandrescu, S., et al. (2022). Endothelial GNAQ p.R183Q increases ANGPT2 (angiopoietin-2) and drives formation of enlarged blood vessels. *Arteriosclerosis, Thrombosis, and Vascular Biology,**42*(1), e27–e43. 34670408 10.1161/ATVBAHA.121.316651 PMC8702487

[13] Couto, J. A., Huang, L., Vivero, M. P., Kamitaki, N., Maclellan, R. A., Mulliken, J. B., et al. (2016). Endothelial cells from capillary malformations are enriched for somatic GNAQ mutations. *Plastic and Reconstructive Surgery*, *137*(1), 77e–82e. 26368330 10.1097/PRS.0000000000001868 PMC5242181

[14] Wetzel-Strong, S. E., Galeffi, F., Benavides, C., Patrucco, M., Bullock, J. L., & Gallione, C. J. (2023). Developmental expression of the Sturge-Weber syndrome-associated genetic mutation in Gnaq: A formal test of Happle’s paradominant inheritance hypothesis. *Genetics*. 10.1093/genetics/iyad077 37098137 10.1093/genetics/iyad077 PMC10894004

[15] Schrenk, S., Bischoff, L. J., Goines, J., Cai, Y., Vemaraju, S., Odaka, Y., et al. (2023). MEK inhibition reduced vascular tumor growth and coagulopathy in a mouse model with hyperactive GNAQ. *Nature Communications,**14*(1), 1929. 37024491 10.1038/s41467-023-37516-7 PMC10079932

[16] Nasim, S., Bichsel, C., Dayneka, S., Mannix, R., Holm, A., Vivero, M., et al. (2024). MRC1 and LYVE1 expressing macrophages in vascular beds of GNAQ p.R183Q driven capillary malformations in Sturge Weber syndrome. *Acta Neuropathologica Communications,**12*(1), 47. 38532508 10.1186/s40478-024-01757-4 PMC10964691

[17] Nasim, S., Bichsel, C., Pinto, A., Alexandrescu, S., Kozakewich, H., & Bischoff, J. (2024). Similarities and differences between brain and skin GNAQ p.R183Q driven capillary malformations. *Angiogenesis*, *27*(4), 931–941. 39343803 10.1007/s10456-024-09950-8 PMC12866883

