# Supplementary material for: Vascular Organoids Derived from Capillary malformation-induced Pluripotent Stem Cells Exhibit Disease-Relevant Phenotypes
Source: Stem Cell Rev Rep. 2025 Sep 25;22(1):668–75. doi: 10.1007/s12015-025-10984-8 (PMC12795959; doi:10.1007/s12015-025-10984-8)
Supplement: Supplementary file 1 [file 12015_2025_10984_MOESM1_ESM.docx]

Supplementary Figure 1: Vascular morphological phenotypes of CTL and CM VOs.  Cross-sections in z position-series from two CTL and two CM VOs were shown. Each VO with z ranges of 100 – 600 um was shown, presenting about 70% of total thickness of the VO. Geen: CDH5 (a.k.a. CD144); Red: aSMA. Dark blue: DAPI nuclei staining. Overlay images were presented.
